# f-Gintropy: An Entropic Distance Ranking Based on the Gini Index

**DOI:** 10.3390/e24030407

**Published:** 2022-03-14

**Authors:** Tamás Sándor Biró, András Telcs, Máté Józsa, Zoltán Néda

**Affiliations:** 1 Wigner Research Centre for Physics, Konkoly-Thege M. 29-33, 1121 Budapest, Hungary; telcs.andras@wigner.hu; 2 Department of Physics, Babeş-Bolyai University, Str. M. Kogalniceanu 1, 400084 Cluj-Napoca, Romania; mate.jozsa@ubbcluj.ro (M.J.); zoltan.neda@ubbcluj.ro (Z.N.); 3 Department of Computer Science and Information Theory, Budapest University of Technology and Economics, Műegyetem rkp. 3, 1111 Budapest, Hungary

**Keywords:** entropy, Gini index, gintropy

## Abstract

We consider an entropic distance analog quantity based on the density of the Gini index in the Lorenz map, i.e., gintropy. Such a quantity might be used for pairwise mapping and ranking between various countries and regions based on income and wealth inequality. Its generalization to f-gintropy, using a function of the income or wealth value, distinguishes between regional inequalities more sensitively than the original construction.

## 1. Introduction

The study of social and economic inequality seems to belong to social sciences and humanities. However, based mostly on economic and econophysics studies, analyses of income and wealth data in terms of mathematical quantities such as the Lorenz curve [1] and the related Gini index [2,3,4] are trying to grasp an ”objective” measure of overall inequality in distributions [5,6,7,8,9].

Given that most interesting distributions have infinite support, the norms on their space are not equivalent. That is why there are numerous measures of distance between distributions. Clearly, the Kullback–Leibler divergence is the most popular one. It is well known that to produce a good estimate of the KL divergence using a limited sample is not easy. That is one of the reasons why several generalization and particular alternatives have been developed. Some are for a specific family of distributions [10,11]; others are better suited to a specific field [12,13]. Our work falls into the latter category, aiming to propose a divergence well adapted to economic or wealth inequalities. The adaptation and generalization of divergences should meet certain criteria The reader can find an overview and new advances in [14,15] in that direction. More recent developments can be found in [16,17,18,19,20], and a recent review in [21].

Certainly, the Gini index is only one among many possible approaches and does not replace the study of the distributions themselves [22]. In this sense, the density of the Gini index in the Lorenz curve—the gintropy—whose precise definition will be given below, includes the total information on the probability density function (PDF) of such distributions.

In an earlier work we introduced this quantity called gintropy [23] and demonstrated its entropy-like properties: for all income PDFs it is non-negative, has a single maximum exactly at the average value and shows overall the corresponding convexity in terms of the cumulative rich population as well as in terms of the cumulative wealth. Based on this property, we propose that gintropy may better reflect the inequality concentrated on the middle class with average income for which much more statistical data are available than for the richest segment of the income and wealth distributions.

In this paper we attempt to generalize this concept to a gintropic distance measure between two PDFs, making it possible to draw a distance graph between countries with varying inequality. By doing so we also investigate the generalization potential in replacing the obvious variable, the income value *x*, with its monotonic function, f(x). That provides the analyzers with some freedom in using differently weighted *f-gintropy* concepts, e.g., using $x^{2}$ or any other quantity behaving coherently with the original one but possibly magnifying some effects. Indeed, we observe a finer distinguishing power by using functions of *x*, describe in a later section.

This article is constructed as follows: First, we review the definitions of gintropy and f-gintropy, controlling their entropy-like properties. Then we construct the f-gintropic divergence measure in analogy to the entropic divergence and study its basic mathematical properties. Finally, based on available data on regional and national income distributions, we develop an inequality distance map. We suggest using these maps as a ”first sight” tool for judging the real effect of inequalities on the population parts near the average.

## 2. Gintropy, f-Gintropy and Gintropic Divergence

The Gini index is a number between zero and one characterizing the inequality inherent in income and wealth distributions. Its definition,

(1) $$G\;\equiv \;\frac{\langle \left|x-y\right|\rangle }{\langle x+y\rangle }\;=\;\frac{\int_{0}^{\infty }\int_{0}^{\infty }\left|x-y\right|P\left(x\right)P\left(y\right)dxdy}{\int_{0}^{\infty }\int_{0}^{\infty }\left(x+y\right)P\left(x\right)P\left(y\right)dxdy},$$

can be used for deriving expressions of *G* in terms of cumulative distributions of the population and the wealth or income possessed by them. These cumulatives,

(2) $$\bar{C}\left(x\right)\;\equiv \;\int_{x}^{\infty }P\left(x\right)dx,\;\bar{F}\left(x\right)\;\equiv \;\frac{1}{\langle x\rangle }\int_{x}^{\infty }xP\left(x\right)dx,$$

plotted against each other present the Lorenz curve in the $\bar{F}-\bar{C}$ plane. This curve is restricted to the unit square, since $\bar{C}\left(0\right)=1$, $\bar{F}\left(0\right)=1$ are the total normalized values, while $\bar{C}\left(\infty \right)=0$ and $\bar{F}\left(\infty \right)=0$ both vanish.

The gintropy, $\sigma \equiv \bar{F}-\bar{C}$, occurs as a density of the Gini index in this map and shows entropy-like features. Based on this, to any probability density function (PDF), such as P(x), one may construct an individual gintropy σ(x). It is interesting to note that the half of the Gini index also can be viewed as the integral of gintropy over any of the cumulatives:

(3) $$G/2\;=\;\int_{0}^{1}\sigma d\bar{C}\;=\;\int_{0}^{1}\sigma d\bar{F}.$$

As such the G/2 value represents the area between the Lorenz curve and the diagonal in the $\bar{C}-\bar{F}$ plane.

The function $\sigma (\bar{C})$ is non-negative, and shows a definite convexity. Furthermore σ(x) is maximal at $x=\langle x\rangle$. Indeed, for several common PDFs $\sigma (\bar{C})$, it resembles entropy formulas suggested so far. For example, for an exponential PDF the classical formula, $\sigma =-\bar{C}\ln\bar{C}$, arises; for a Pareto distribution P(x), we obtain a Tsallis q-entropy formula $\sigma \left(\bar{C}\right)=\left({\bar{C}}^{q}-\bar{C}\right)/\left(1-q\right)$. The particular value, q=2, resembles the entanglement entropy, $S=\mathrm{Tr}(\rho -\rho^{2})$ with ρ being the density operator, used in some quantum computing calculations. These analogies are elaborated in [23].

In the present paper we attempt to generalize this construction to a possible use of another quantity’s cumulative tail more integral than the original variable, *x*. Moreover, based on this, we construct an entropic divergence measure for the purpose of using it as an ”inequality distance” between PDFs based on their generalized Lorenz curve behavior, comprised in the quantity *f-gintropy*.

Let P(x) be an absolute continuous PDF over [0,∞). To begin with, we consider only *P* and do not use any subscript related to it. Later, when considering two different PDFs and constructing their f-gintropic distance measure, we shall retain such indices.

Let *f* be a monotonic, non-negative differentiable function with $f\left(0\right)=0.$ Assume that the expectation value,

(4) $$\langle f\rangle =\int_{0}^{\infty }f\left(x\right)P\left(x\right)dx<\infty ,$$

exists. Let the truncated f-moment be defined as

(5) $${\bar{F}}_{f}\left(x\right)\;\equiv \;\frac{1}{\langle f\rangle }\;\int_{x}^{\infty }f\left(y\right)P\left(y\right)dy$$

and we use the original tail-cumulative $\bar{C}\left(x\right)$ defined in Equation (2). Inverting the above definitions, one notes that $d\bar{C}/dx=-P\left(x\right)$ and $d{\bar{F}}_{f}/dx=-f\left(x\right)P\left(x\right)/\langle f\rangle$. Hence, also $d{\bar{F}}_{f}/d\bar{C}=f\left(x\right)/\langle f\rangle$.

The *f*-Gini index is constructed based on the geometry of the Lorenz curve. First, one easily proves that

(6) $$\int_{0}^{1}{\bar{F}}_{f}d\bar{C}\;+\;\int_{0}^{1}\bar{C}d{\bar{F}}_{f}\;=\;1.$$

Second, the corresponding Gini index is defined as the difference between these two integrals

(7) $$G_{f}\;\equiv \;\int_{0}^{1}{\bar{F}}_{f}d\bar{C}\;-\;\int_{0}^{1}\bar{C}d{\bar{F}}_{f}.$$

Let us first prove the statement about the sum in Equation (6). The first integral can be expanded as

(8) $$\int_{0}^{1}{\bar{F}}_{f}d\bar{C}\;=\;\int_{0}^{\infty }{\bar{F}}_{f}\left(x\right)P\left(x\right)dx\;=\;\int_{0}^{\infty }dxP\left(x\right)\int_{x}^{\infty }dyP\left(y\right)\frac{f(y)}{\langle f\rangle },$$

while the second similarily as

(9) $$\int_{0}^{1}\bar{C}d{\bar{F}}_{f}\;=\;\int_{0}^{\infty }\bar{C}\left(x\right)\frac{f(x)}{\langle f\rangle }P\left(x\right)dx\;=\;\int_{0}^{\infty }dxP\left(x\right)\frac{f(x)}{\langle f\rangle }\;\int_{x}^{\infty }P\left(y\right)dy.$$

The second integral can be rewritten by exchanging the dummy integration variables *x* and *y* among each other,

(10) $$\int_{0}^{1}\bar{C}d{\bar{F}}_{f}\;=\;\int_{0}^{\infty }dyP\left(y\right)\frac{f(y)}{\langle f\rangle }\;\int_{y}^{\infty }P\left(x\right)dx.$$

Then we interchange the order of integration to arrive at

(11) $$\int_{0}^{1}\bar{C}d{\bar{F}}_{f}\;=\;\int_{0}^{\infty }dxP\left(x\right)\int_{0}^{x}dyP\left(y\right)\frac{f(y)}{\langle f\rangle }.$$

Indeed, the sum of Equations (8) and (11) becomes

(12) $$\int_{0}^{1}{\bar{F}}_{f}d\bar{C}\;+\;\int_{0}^{1}\bar{C}d{\bar{F}}_{f}\;=\;\int_{0}^{\infty }dxP\left(x\right)\left[\int_{0}^{x}dyP\left(y\right)\frac{f(y)}{\langle f\rangle }+\int_{x}^{\infty }dyP\left(y\right)\frac{f(y)}{\langle f\rangle }\right].$$

Here, obviously the two integrals inside the square bracket can be unified in a single integral proving that

(13) $$\int_{0}^{1}{\bar{F}}_{f}d\bar{C}\;+\;\int_{0}^{1}\bar{C}d{\bar{F}}_{f}\;=\;\int_{0}^{\infty }dxP\left(x\right)\int_{0}^{\infty }dyP\left(y\right)\;\frac{f(y)}{\langle f\rangle }\;=\;\int_{0}^{\infty }dxP\left(x\right)\frac{\langle f\rangle }{\langle f\rangle }\;=\;1.$$

Inspecting a general Lorenz curve drawn in the unit square on the $\bar{C}-{\bar{F}}_{f}$ plane, it is obvious that the area between the curve and diagonal represents the half of the Gini index, defined in Equation (7), as the difference of integrals.

We are interested further in its equivalent forms. We would like to view $G_{f}$ also as an expectation value of something. We utilize the forms (8) and (9) to write

(14) $$G_{f}\;=\;\int_{0}^{\infty }dxP\left(x\right)\int_{x}^{\infty }dyP\left(y\right)\;\frac{f(y)-f(x)}{\langle f\rangle }.$$

Exchanging the integration variables again and reordering the integrations, an equivalent expression emerges

(15) $$G_{f}\;=\;\int_{0}^{\infty }dxP\left(x\right)\int_{0}^{x}dyP\left(y\right)\;\frac{f(x)-f(y)}{\langle f\rangle }.$$

Now we fix a few requirements for the function f(x). To map ${\bar{F}}_{f}$ between zero and one f(x),≥0 is necessary. To interpret the f-Gini index as an expectation of an absolute value of difference, the strict monotonity property, f(x)>f(y) for x>y and equivalently f(x)<f(y) for x<y is required. Fulfilling these requirements, one obtains that $G_{f}$ is also the half of the sum of Equations (14) and (15):

(16) $$G_{f}\;=\;\frac{\langle \left|f(x)-f(y)\right|\rangle }{\langle \left|f(x)+f(y)\right|\rangle }.$$

This agrees with the original definition for f(x)=x and follows its form ready for data evaluation.

Based on the above, it is natural to introduce f-gintropy as

(17) $$\sigma_{f}\;\equiv \;{\bar{F}}_{f}-\bar{C}.$$

This quantity has the following properties, based on the already discussed restrictions on f(x), i.e., f(x)≥0 and $f^{\prime }\left(x\right)>0$:

- It is never negative, $\sigma_{f}\geq 0$.
- It is zero for x=0 and x=∞ only, or at $\bar{C}=0$ and $\bar{C}=1$.
- It is maximal at the $x=x_{m}$ value, fulfilling $f\left(x_{m}\right)=\langle f\rangle$.
- It has a single maximum only, either as a function of *x* or $\bar{C}$ or ${\bar{F}}_{f}$.
- The f-gintropy, $\sigma_{f}$, like the entropy, is everywhere concave on $\bar{C}$ and ${\bar{F}}_{f}$.

We give a short analysis of the above statements as follows.

(18) $$\sigma_{f}\left(x\right)=\frac{1}{\langle f\rangle }\;\int_{x}^{\infty }dyP\left(y\right)\left[f\left(y\right)-\langle f\rangle \right]$$

is positive if $f\left(x\right)>\langle f\rangle$, since for all y>x in the above integral one has $f\left(y\right)>f\left(x\right)>\langle f\rangle$. For the opposite choice of *x*, when $f\left(x\right)\leq \langle f\rangle$, we rewrite the gintropy expression as

(19) $$\sigma_{f}\left(x\right)=\left[1-\int_{0}^{x}dyP\left(y\right)\frac{f(y)}{\langle f\rangle }\right]\;-\;\left[1-\int_{0}^{x}dyP\left(y\right)\right]\;=\;\frac{1}{\langle f\rangle }\;\int_{0}^{x}dyP\left(y\right)\left[\langle f\rangle -f\left(y\right)\right].$$

Again, due to the monotonity of *f* for all y≤x in the integration range, we have $f\left(y\right)\leq f\left(x\right)\leq \langle f\rangle$. That finishes the proof of $\sigma_{f}\geq 0$. It is also obvious from the above expressions that equality occurs only if using x=0 or x→∞.

Let us turn now to the discussion of concavity. For that purpose we calculate the first and second derivatives of gintropy w.r.s.p to $\bar{C}$ and ${\bar{F}}_{f}$. These are obtained in turn from the *x*-derivative,

(20) $$\frac{d\sigma_{f}}{dx}\;=\;\frac{d{\bar{F}}_{f}}{dx}-\frac{d\bar{C}}{dx}\;=\;\left(1-\frac{f(x)}{\langle f\rangle }\right)P\left(x\right).$$

This quantity changes sign exactly where $f\left(x_{m}\right)=\langle f\rangle$ and nowhere else.

The $\bar{C}$- and ${\bar{F}}_{f}$-derivatives can be obtained using the $d\bar{C}/dx$ and $d{\bar{F}}_{f}/dx$ values shown earlier in the text. One calculates

(21) $$\frac{d\sigma_{f}}{d\bar{C}}\;=\;\frac{f(x)}{\langle f\rangle }-1,\;\mathrm{and}\;\frac{d{}^{2}\sigma_{f}}{d{\bar{C}}^{2}}\;=\;-\frac{f^{\prime }\left(x\right)}{P\left(x\right)\langle f\rangle }.$$

Due to the strict monotonity of *f*, we have $f^{\prime }\left(x\right)>0$, and therefore, the second derivative of f-gintropy is always negative. We similarly arrive at the same conclusion in terms of $F_{f}$:

(22) $$\frac{d\sigma_{f}}{d{\bar{F}}_{f}}\;=\;1-\frac{\langle f\rangle }{f(x)},\;\mathrm{and}\;\frac{d{}^{2}\sigma_{f}}{d{\bar{F}}_{f}^{2}}\;=\;-\frac{f^{\prime }\left(x\right)\langle f\rangle }{P\left(x\right)f^{3}\left(x\right)}\;<\;0.$$

Let us recall that the Gini index and its generalization can be written as an integral over the gintropy, $\sigma_{f}$.

We built the f-gintropic divergence measure following the construction pattern of the Kullback–Leibler divergence. It is also in line with the idea how Csiszár’s f-divergence has been introduced [24,25]. For recent advances of the f-divergence and its estimate see, e.g., [26,27]. The main difference is that while the *f-entropy* due to Csiszár replaces the logarithm of the probability with a general function in the entropy formula, we generalize the tail cut integral of the income to a function of it. The latter function will be restricted, as discussed later.

Instead of using the PDFs, we suggest applying a similar formula to the gintropies. Since the original gintropy function is not normalized, we use its normalized version

(23) $$\hat{\sigma }\left(x\right)\;\equiv \;\frac{\sigma (x)}{\langle \sigma \rangle }$$

with

(24) $$\langle \sigma \rangle \;=\;\int_{0}^{\infty }\sigma \left(x\right)\;P\left(x\right)dx\;=\;\int_{0}^{1}\sigma d\bar{C}=G/2.$$

Then $\hat{\sigma }\left(\bar{C}\right)$ behaves as a PDF mathematically: its integral over the possible $\bar{C}$-range is unity, and it is everywhere non-negative. Analogous to the definition of classical entropic divergences, we propose a Kullback–Leibler type Gini divergence between the distributions $P_{1}\left(x\right)$ and $P_{2}\left(x\right)$ as

(25) $${\mathrm{KLGD}}_{f}\left(P_{1}\left|\right|P_{2}\right)=\int_{0}^{1}{\hat{\sigma }}^{\left(2\right)}\log\left(\frac{{\hat{\sigma }}^{\left(2\right)}}{{\hat{\sigma }}^{\left(1\right)}}\right)\;d\bar{C}.$$

It is straightforward to generalize further by using a general convex function *s* instead of the logarithm with $s\left(1\right)=0$ and $s^{\prime \prime }<0$. One immediately realizes that ${\mathrm{KLGD}}_{s,f}\left(P_{1}\left|\right|P_{2}\right)=0$ iff $P_{1}=P_{2}$ almost surely and ${\mathrm{KLGD}}_{s,f}\left(P_{1}\left|\right|P_{2}\right)\geq 0$ in general, based on the convexity inherent in the definition.

Analogous to the definition of mutual information, the mutual gintropy, MG, can also be introduced as the gintropic divergence between the joint distribution and the product of the subdistributions:

(26) $$MG\left(P_{1};P_{2}\right)=KLGD_{s,f}\left(P_{1}⊗P_{2}\left|\right|P_{1,2}\right).$$

## 3. Example: Gintropic Distance Ranking of Income Data

**Figure 1:**
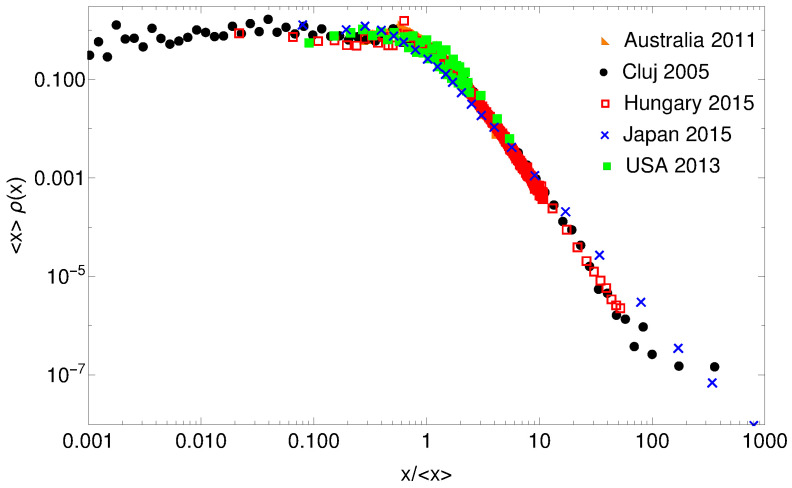
Probability density function for the distributions of the normalized income for some countries and geographical regions. The income for each region is normalized to the respective average value. Please note that we use log-log scales.

We will exemplify the use of gintropic distance reconsidering the income distribution data studied in [28]. Data for Australia (2011), Hungary (2015), Japan (2015), USA (2013) and Cluj county, Romania (2005) are used. These are the same data as the ones used in [28], and they were selected for statistical studies due to their free availability and higher resolutions in the income intervals, allowing for a precise construction of relevant quantities. The data for Cluj county are exhaustive from a social security database, containing the income of all registered employee from that region [29]. To use a common scale and to collapse the distributions as much as possible, we consider for each case the normalized income, i.e., income relative to the average value. The probability density functions of these distributions for the whole normalized income ranges are plotted in Figure 1. They look very similar on log-log scale, and only the data for Japan seems to have a different scaling in the high income region. On such a log-log representation, the probability density functions of normalized income for Hungary and Cluj county (Romania) seem to collapse perfectly, which is definitely a consequence of the closely related economic history of these two countries.

**Figure 2:**
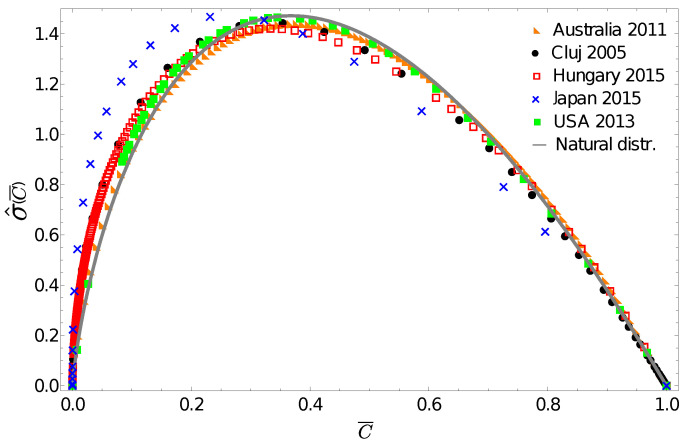
Normalized gintropy $\hat{\sigma }\left(\bar{C}\right)$ calculated from the income distribution data in comparison with the one expected for the natural distribution (27).

One can learn from population biology that in many cases the use of the probability density function is not the best methodology to illustrate the relevant differences in abundance [30]. For heavy-tailed distributions, the generally used log-log scales (such as in Figure 1) is appropriate to illustrate scaling, but it does not offer relevant information for those parts of the distribution where the majority of the individuals are. Therefore in illustrating these differences, instead of the mathematically well-defined probability density function, a special frequency histogram, the Preston plot [31], is used. When illustrating social inequalities in form of income, we are in a similar situation. As an alternative to the probability density, from the income distribution data one can construct a normalized gintropy $\hat{\sigma }\left(\bar{C}\right)=\sigma \left(\bar{C}\right)/\langle \sigma \rangle$ by estimating numerically both cumulative functions, $\bar{C}\left(x\right)$ and $\bar{F}\left(x\right)$, from the data themselves. In contrast to the probability density function, this quantity will peak in the vicinity of the average income, i.e., characterizing the income regions where the majority of the population is to be found. Figure 2 presents these gintropy functions in comparison to the one expected for the natural (exponential) distribution:

(27) $${\hat{\sigma }}_{nat}\left(\bar{C}\right)=-4\;\bar{C}\;\ln\left(\bar{C}\right)$$

Figure 2 again reveals the fact that the income distribution for Japan seems to be very different, and surprisingly, we see that the gintropy curve for Australia is the closest to the one for the natural distribution. The gintropies, $\hat{\sigma }\left(\bar{C}\right)$, for Hungary and Cluj county are also quite close to that of the natural distribution. All these results confirm again what we have known for a long time: both income and wealth distributions tend to have an exponential shape when restricted to the middle class of a society [32,33].

Gintropic distances can be calculated using the well-known Kullback–Leibler divergences for the $\hat{\sigma }\left(\bar{C}\right)$ functions.

(28) $$\mathrm{KLGD}(P_{i}\left|\right|P_{j})=\int_{0}^{1}{\hat{\sigma }}_{i}\left(\bar{C}\right)\log\left(\frac{{\hat{\sigma }}_{i}\left(\bar{C}\right)}{{\hat{\sigma }}_{j}\left(\bar{C}\right)}\right)\;d\bar{C}$$

Approximating the above integral from the interpolated experimental data for $\hat{\sigma }\left(\bar{C}\right)$, we thus determine a quantity that characterizes the gintropic differences between income distributions among different countries. In Table 1 we indicate these values and also consider the distance relative to the normalized gintropy which is characteristic to the natural distribution ${\hat{\sigma }}_{nat}\left(\bar{C}\right)$. This table again suggests that while focusing on the relevant (larger) part of the society, the income distribution in Australia and the USA are close to the expected natural distribution. On the other hand, Hungary, Romania and Japan seem to be different: an interesting and unexpected result. Such a methodology based on the gintropy instead of the commonly used probability density function could definitely be useful in cases where one prefers to group countries in clusters according to their most abundant income categories.

One can now go further and test whether the $\hat{\sigma }\left(\bar{C}\right)$ gintropy, associated to f(x)=x, can be fitted with the gintropy for the Tsallis–Pareto distribution with 〈x〉=1:

(29) $$P\left(x\right)=\frac{1}{q}{\left(1+\frac{1-q}{q}x\right)}^{-\frac{2-q}{1-q}}$$

The corresponding normalized gintropy is proportional to the Tsallis q-entropy form [23]:

(30) $${\hat{\sigma }}_{q}\left(\bar{C}\right)=2\;\frac{1+q}{1-q}\left({\bar{C}}^{q}-\bar{C}\right).$$

**Figure 3:**
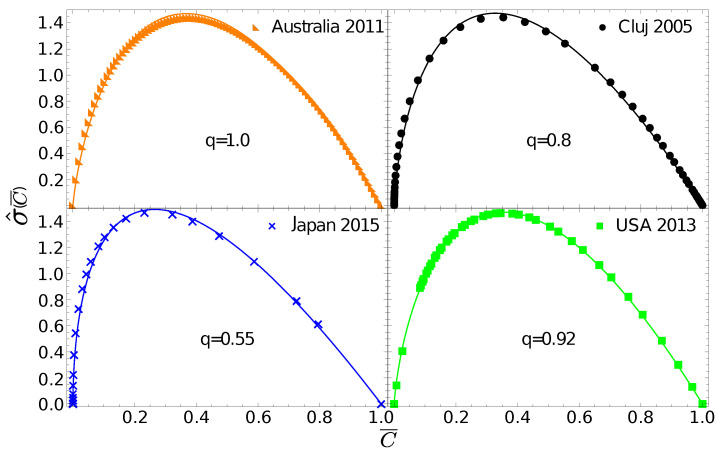
Gintropy for different regions fitted with the one derived for the Tsallis–Pareto distribution ${\hat{\sigma }}_{q}\left(\bar{C}\right)$ (30). In the figures we illustrate the best best fit and also give the best-fit parameter, *q*. There is no separate panel for Hungary since the experimental gintropy for Hungary and Cluj are very close, as already seen in Figure 2.

In Figure 3 we fit the experimental gintropy curves with the one given in Equation (30). The best fit parameter, q=1, for Australia again suggests that in this case the natural distribution is relevant since Equation (30) in the limit q=1 leads to Equation (27). For Cluj county and Hungary, the best fit is obtained with q=0.8, for the USA we get q=0.92, and for Japan we obtain q=0.55.

One can now use the above discussed income distributions to exemplify the application potential of the f-gintropy. We choose a simple convex function $f\left(x\right)=x^{2}$ and construct the corresponding ${\hat{\sigma }}_{f}\left(\bar{C}\right)$ normalized f-gintropy for all the income distributions. Such a choice is justified if: (i) instead of income one considers some other socioeconomic metrics that depend on its square or (ii) if we intend to amplify differences at large income values. In this latter case, any convex-shaped f(x) function can serve this purpose.

**Figure 4:**
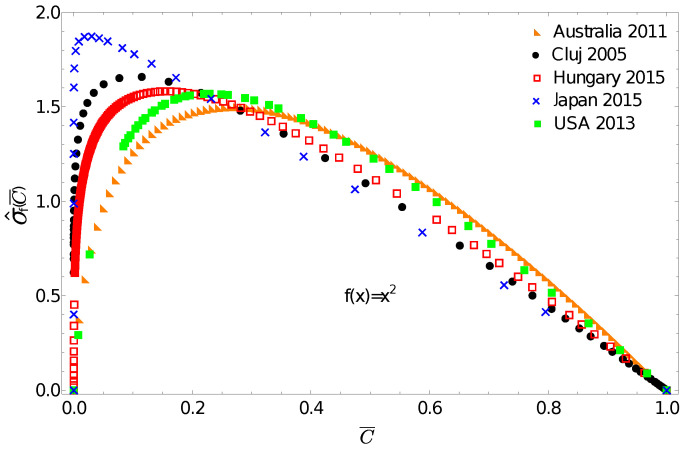
Normalized f-gintropy ${\hat{\sigma }}_{f}\left(\bar{C}\right)$ with $f\left(x\right)=x^{2}$ calculated from the income distribution data. Note the more evident separation of the studied geographical regions.

The obtained curves are plotted in Figure 4. A first immediate consequence of using the f-gintropy instead of the usual gintropy is that the ${\hat{\sigma }}_{f}\left(\bar{C}\right)$ curves become more strongly separated. Japan and Australia are again the two extremes. In such a representation, one can distinguish between the income distributions in Hungary and Cluj county that appeared to be very close before. A Kullback–Leibler distance matrix, similar to the one shown in Table 1, is now constructed for the f-gintropy with the $f\left(x\right)=x^{2}$ choice. To proceed, we used a second order interpolation method to estimate the Kullback–Leibler divergence for the f-gintropy in the ${\hat{\sigma }}_{f}\left(\bar{C}\right)$ representation. The results are given in Table 2. According to the values from Table 2, Australia and the USA form a clear cluster, and similarly Hungary with Cluj county and Japan continues to be in a separate cluster. While the similarities between Australia and the USA together with the ones between Hungary and Romania are easy to interpret, Japan’s position seems to be more surprising. These results might confirm some earlier hypotheses according to which the Japanese taxation and redistribution system is perfectly balanced, neither highly redistributive nor too capitalistic [34]. Interestingly, their income redistribution policies seem to be closer to those in former socialist countries rather than the ones with consecrated free market economies. In such a view, the results obtained here make sense.

## 4. Conclusions

In conclusion, we have constructed a generalization of the usual Lorenz-curve based entropy similar to the concept, gintropy, developed by us earlier. This generalization involves weighting functions more general than the original income values, replacing *x* by f(x), the only requirement being its monotonicity and non-negativity. Entropic properties of such a generalization were demonstrated.

A Kullback–Leibler type entropic divergence was used to inspect clusterization among income distribution PDFs stemming from different regional groups. In the example of detailed data from five different regions (Japan, Australia, USA, Hungary and Cluj) we have demonstrated that: (i) the gintropy emphasizes data near the average income, and (ii) the use of weight functions rising steeper than *x* enhances differences not otherwise seen. The former result is practical, since data about extreme high incomes and wealth are usually insufficient or unreliable. The latter may help resolve close looking data in the future, as a change of viewing angle can resolve contours appearing to be the same while they are not.

## Figures and Tables

**Table 1:** Gintropic distances for income distributions calculated by using the (28) gintropic Kullback–Leibler divergences. We also indicate this distance relative to the natural distribution.

$\times 10^{-3}$	**Natural**	Australia	USA	Cluj	Hungary	Japan
**Natural**	0	0	0.4	2.9	2.9	17
Australia	0	0	0.4	2.9	2.9	17
USA	0.4	0.4	0	1.1	1.1	13
Cluj	3	3	1.1	0	0	6.4
Hungary	3	3	1.1	0	0	6.4
Japan	19	19	14	6.8	6.8	0

**Table 2:** f-gintropic distances for income distributions calculated using the (28) generalized Kullback–Leibler divergences for $f\left(x\right)=x^{2}$.

$\times 10^{-3}$	Australia	USA	Cluj	Hungary	Japan
Australia	0	1.8	36	19	62
USA	1.7	0	27	13	50
Cluj	48	39	0	3.8	4
Hungary	22	16	3.5	0	14
Japan	86	74	4.3	17	0

## Data Availability

Not applicable.
